# Making Genomic Data Actionable in Electronic Health Records: Practical Viewpoint on Standards, Governance, and Implementation

**DOI:** 10.2196/99130

**Published:** 2026-10-21

**Authors:** Styliana Menelaou, Eirini C Schiza, Marios Tomazou, Andreas Neocleous, Anastasis Oulas, Menelaos Pipis, Andreas Menikou, Athina Theodosiou, Maria Papaioannou, Carolina Sismani, Theodoros Kyprianou, George M Spyrou, Christos N Schizas

**Affiliations:** 1 Department of Bioinformatics, Cyprus Institute of Neurology and Genetics, 6 Iroon Avenue, 2371 Ayios Dometios, Nicosia, 2371, Cyprus, 357 99045197; 2 University of Cyprus Medical School, Nicosia, Cyprus; 3 National eHealth Authority of Cyprus, Nicosia, Cyprus; 4 Department of Neuropathology, Cyprus Institute of Neurology and Genetics, Nicosia, Cyprus; 5 Department of Cytogenetics and Genomics, Cyprus Institute of Neurology and Genetics, Nicosia, Cyprus; 6 Biomedical Engineering Research Centre (BERC), University of Cyprus, Nicosia, Cyprus; 7 Medical School University of Nicosia, Nicosia, Cyprus

**Keywords:** electronic health records, EHRs, genomics, precision medicine, personalized medicine, interoperability, HL7 FHIR, clinical decision support systems, CDSS, pharmacogenomics, European Health Data Space, EHDS

## Abstract

Electronic health records (EHRs) are central to patient-centered care. However, many current EHR systems do not represent, exchange, or reuse genomic findings in structured, interoperable, and clinically actionable forms. Instead, genomic results are often retained in static reports. This limits their use in routine clinical care and clinical decision support. It also restricts their future reinterpretation as scientific knowledge evolves, as well as their reuse for research within appropriate governance frameworks. To support precision and personalized medicine, we advocate that clinically relevant genomic findings should be represented within EHRs as structured, phenotype-linked, interoperable, and versioned data. Raw and intermediate genomic files, however, should remain in appropriately governed laboratory or genomics repositories. This viewpoint is intended for health policymakers, health informatics leaders, EHR developers, research and clinical genomics laboratories, and clinicians involved in implementing genomics into routine care. It is informed by selected published literature and relevant technical standards. It is also informed by operational experience at the Cyprus Institute of Neurology and Genetics and multidisciplinary discussions from the Gen2EHR Implementation Guidelines Workshop conducted within the Extended EHR@EU Data Space and European Health Data Space for Secondary Use @CY initiatives. We examine technical, organizational, clinical, ethical, legal, and governance challenges, including inconsistent reporting formats and unclear clinical responsibility. We propose a staged, workshop-informed implementation pathway for evaluation within the European Health Data Space. The pathway combines Health Level 7 Fast Health Interoperability Resources Genomics Reporting with complementary standards and terminologies. It also includes transitional dual PDF and structured Fast Health Interoperability Resources reporting, governance for access, version control, reinterpretation, and clinical responsibility, as well as multidisciplinary pilot validation in real diagnostic workflows. The clinical effectiveness and transferability of the proposed pathway across European health systems remain to be established.

## Introduction

Advances in next-generation sequencing technologies have expanded the clinical availability of genomic testing, creating new opportunities for precision and personalized medicine [1,2]. Genomic findings now support disease diagnosis, prognosis, treatment selection, and pharmacogenomics-informed prescribing [3-5]. However, despite ongoing European initiatives, practical guidance for integrating clinically relevant genomic findings into electronic health records (EHRs) remains limited [6,7].

EHRs are structured, continuously updated records of an individual’s health information throughout life [8,9]. They support patient management, improve care coordination, strengthen clinical decision-making, and facilitate public health and biomedical research [10,11]. They also enable the integration of clinical decision support systems (CDSSs) and other intelligent tools that support the workforce, contributing to safer, more efficient, and more personalized health care [2,11]. Their effectiveness depends on interoperability, which allows health information to be exchanged and interpreted consistently across different health care systems [8,12]. This provides the foundation for integrating emerging data types, including genomic information [13-15].

Within Europe, several initiatives are supporting this transition, including the Extended EHR@EU Data Space (Xt-EHR) Joint Action and the European Health Data Space for Secondary Use @CY (CY-EHDS-2nd) project [16,17]. Together, they contribute to the implementation of the European Health Data Space (EHDS) [18]. Xt-EHR focuses on the primary use of health data for clinical care and prevention, whereas CY-EHDS-2nd supports the secondary use of health data for research and innovation. Both initiatives promote secure, interoperable, and citizen-centered exchange of health information across Member States.

Despite these advances, genomic findings are still commonly returned to EHRs as static reports that are difficult to exchange, update, and reuse within modern health care systems [6,7,19,20]. This limits their clinical value and hinders the broader implementation of genomics into routine patient care. To fully realize the potential of genomic medicine, EHR systems must evolve to support the structured representation, exchange, and reuse of clinically relevant genomic information [1,2,21].

To examine these implementation needs, the National eHealth Authority of Cyprus (NeHA), the University of Cyprus (UCY), and the Cyprus Institute of Neurology and Genetics (CING) organized the Gen2EHR Implementation Guidelines (IG) Workshop under the framework of the Xt-EHR and CY-EHDS-2nd EU-funded projects. The workshop brought together experts from clinical, technical, and health policy backgrounds to discuss the technical requirements, coding systems, and interoperability standards needed for the meaningful integration of genomics into EHRs.

Rather than reviewing all aspects of genomic medicine, in this viewpoint, we draw on selected published literature, relevant technical standards, operational experience with genomic testing and reporting at CING, and multidisciplinary discussions from the Gen2EHR Workshop. We discuss the technical, organizational, clinical, ethical, legal, and governance challenges that currently limit the routine integration of genomics into EHRs. Building on these considerations, we propose a practical roadmap to support implementation across the EHDS.

## Clinical and Research Value of Structured Genomic Findings in EHRs

EHRs provide a longitudinal view of patient information and support multidisciplinary care and clinical decision-making [2,22]. Their value can be further extended when clinically relevant genomic findings are represented as structured, phenotype-linked, interoperable, and versioned data [20]. Because the interpretation of genomic variants may change as scientific knowledge evolves, structured integration also supports variant reinterpretation and allows updated findings to be considered alongside diagnoses, medications, laboratory results, and other clinical information [23,24].

Once available in this form, genomic findings may support clinical decision-making, personalized treatment strategies, and longitudinal patient management [2]. They may also contribute to a learning health system in which knowledge generated through clinical practice and research informs future patient care. However, these benefits depend on high-quality data, clinical validation, and integration into routine workflows [20,24].

A well-established example is pharmacogenomics [25]. When validated pharmacogenomic findings are represented in a structured format within EHRs, they become available for use by applications such as CDSSs. CDSSs can then alert clinicians to gene-drug interactions and genetic variants associated with altered drug response or toxicity. This reduces reliance on manual review of static genomic reports and makes clinically relevant information available within routine workflows [26,27]. However, CDSSs should complement, rather than replace, professional judgment and access to laboratory or clinical genetics expertise.

The value of genomic information also extends beyond direct patient care. Under appropriate legal, ethical, and governance frameworks, harmonized genomic and phenotypic data may support secondary uses such as genome-wide association studies, phenome-wide association studies, and the interpretation of rare genetic variants by comparing patients with similar genomic and clinical profiles [20]. These applications can improve our understanding of disease mechanisms, facilitate variant interpretation, and support future advances in clinical care and research [2,10,28].

Overall, structured, clinically relevant genomic findings have the potential to improve both clinical care and biomedical research. Realizing these benefits, however, requires addressing the implementation challenges discussed in the following section.

## Challenges in Integrating Genomic Findings Into EHRs

Integrating genomic findings into EHRs requires addressing several technical, organizational, clinical, and governance issues. These include data representation, storage, interoperability, workforce readiness, privacy, and the evolving nature of genomic knowledge.

EHRs contain structured, semistructured, and unstructured data. Structured data use standardized, encoded, and machine-readable formats that support interoperability and CDSSs [7,10,29,30]. In contrast, genomic test results are still commonly stored and exchanged as free-text/unstructured reports or PDF files [31,32]. These formats are intended for human interpretation but limit data reuse, interoperability, and integration into clinical workflows [2,7,10]. Semistructured formats, such as JSON and XML, provide an intermediate approach but do not fully address these limitations [10,20,33].

Genomic analysis also produces large and technically complex datasets that most EHRs are not designed to store or process. Therefore, specialized genomic repositories should store raw and intermediate data, while EHRs should contain only clinically relevant findings needed for patient care [20,24,34].

Another challenge is that genomic knowledge evolves over time. Variant interpretation may change as new evidence becomes available. EHR-integrated genomic findings therefore require provenance, date-stamped interpretations, version control, and clear processes for review and update. Responsibilities for reinterpretation, clinical validation, communication of updated findings, and, where appropriate, patient recontact should be clearly defined [20,24,34].

Successful implementation also depends on clinical workflow and workforce readiness. Clinicians need appropriate education to understand the significance and limitations of genomic findings and to communicate uncertain results to patients. Although CDSSs can aid routine care, their effectiveness depends on appropriate clinical training, professional judgment, and collaboration with laboratory and clinical genetics specialists. Their usability, reliability, and integration into routine care should also be carefully evaluated [31,32,35,36].

Genomic information is highly sensitive and may have implications for biological relatives as well as individual patients. Its integration into EHRs raises important privacy, security, ethical, legal, and governance considerations [37,38]. Appropriate governance should address data processing, informed consent where required, role-based access, auditability, data retention, primary and secondary use of genomic information, and responsibilities for returning clinically relevant findings. Furthermore, many variants remain classified as variants of uncertain significance (VUSs), increasing the risk of misinterpretation, overdiagnosis, or unnecessary anxiety [39-41]. Transparent communication and appropriate professional support are therefore essential.

In summary, addressing these challenges requires more than technical system development. It requires standardized representation of clinically relevant findings, appropriate data governance, workforce preparedness, and reliable interoperability across laboratories, genomic repositories, EHR systems, health care organizations, and jurisdictions. The following section discusses the key dimensions of interoperability needed to support the effective integration of genomics into health care systems.

## Dimensions of Interoperability in Health Systems

To effectively address these challenges and facilitate the integration and exchange of genomic data across EHR systems, appropriate regulations and interoperability standards must be established. The Institute of Electrical and Electronics Engineers defines interoperability as “the ability of two or more systems or components to exchange information and to use the information that has been exchanged” [33,42,43]. As portrayed in Figure 1, there are 5 dimensions of interoperability, each addressing the need for standards to facilitate seamless communication between EHR systems, exchange, understand, interpret, and protect patient health information [42].

**Figure 1.**
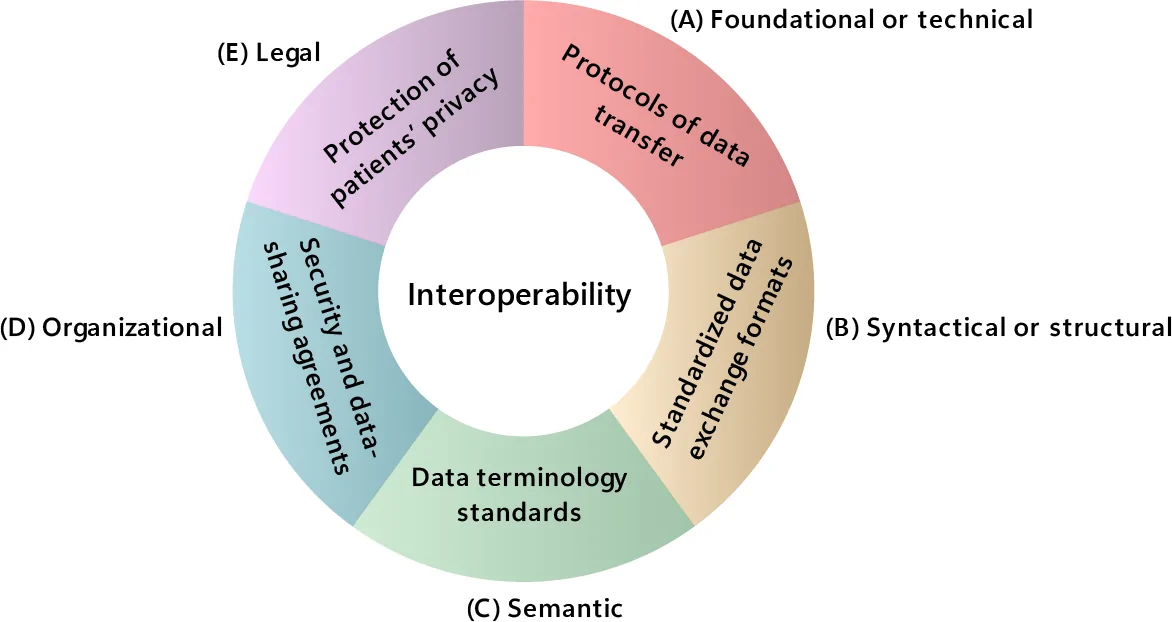
Dimensions of interoperability and examples of supporting standards. This circular diagram illustrates the 5 equally important and interdependent types of interoperability required for the meaningful and secure exchange of health data between systems. These include (A) foundational or technical interoperability, which sets the foundation and standards for creating technical specifications for communication and data exchange between systems (eg, Health Level 7 Fast Health Interoperability Resources); (B) syntactic and structural interoperability, which defines standards for the structure and coding of data so that when data are exchanged from one system to another, their meaning remains unchanged (eg, JSON/XML formats); (C) semantic interoperability, which sets standards for the use of standardized vocabularies, terminologies, and data models to ensure that the information exchanged is maintained, interpreted, and understood in the same way by both sending and receiving systems (eg, Logical Observation Identifiers Names and Codes, Systematized Nomenclature of Medicine); (D) organizational interoperability, which sets standards for governance, policy, social, legal, and organizational issues to enable seamless exchange and use of health information; and (E) legal interoperability, which sets standards for sharing patient data and complying with relevant legal and regulatory requirements, protecting data privacy, security, and intellectual property rights (eg, General Data Protection Regulation) [42].

## Health Level 7 Fast Health Interoperability Resources as a Core Enabler of Genomic Interoperability

When working with genomic data, it is important that EHR systems can communicate effectively with Laboratory Information Systems. As shown in Figure 2, the process usually begins when a clinician submits a *ServiceRequest* form within the EHR system, specifying the genomic test to be performed (eg, whole exome sequencing). A *specimen* (eg, blood sample, saliva, or tissue biopsy) is then collected and forwarded to the laboratory. The lab conducts a genomic analysis to detect relevant variants, which are captured as structured *Observations* (eg, variant, genotype, implication data, etc). These Observations are then aggregated into a *DiagnosticReport* that summarizes the findings. Often, this report is supplemented by files such as PDFs or raw genomic data (eg, variant call files), linked via a *DocumentReference* resource. In cases where the genetic findings suggest a specific clinical action (eg, changing a medication or referring the patient for further testing), a *Task* resource is created to formalize and transmit that recommendation, enabling downstream care teams to act. This entire workflow, from the initial test request to the clinical action, is supported by interoperability standards (see Figure 1), which ensure that data are shared and understood in the same way across different systems [32,44,45].

**Figure 2.**
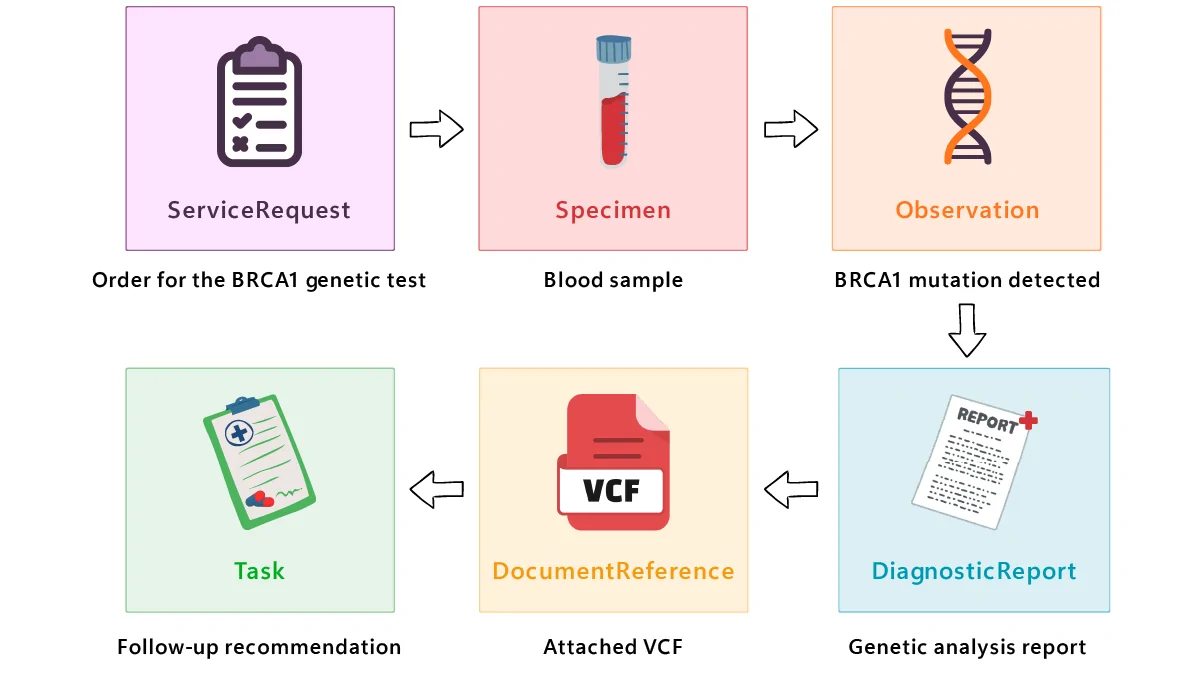
Workflow illustrating the typical steps involved in the bidirectional exchange of genomic data between electronic health records and laboratory information systems. The workflow starts with a test request and ends with a clinical recommendation, showing how structured data and attached files (like variant call files) are shared using Fast Health Interoperability Resources resources. It uses a BRCA1 mutation as an example [45]. BRCA1: breast cancer gene 1; VCF: Variant Call Format.

To enable this workflow in practice, the exchange of genomic data between EHRs and Laboratory Information Systems must be built on a strong technical foundation. This begins with the implementation of basic interoperability standards that allow systems to communicate consistently (see Figure 1A). Traditionally, this has been achieved through standards like HL7 v2 (Health Level 7, version 2), which uses predefined message formats. While still in use, HL7 v2 was not designed with modern genomic data in mind [7,21,32].

Genomic data are inherently complex and voluminous, often requiring the transfer of structured information that includes genetic variants, interpretations, and related files [20]. To support these requirements, HL7 developed FHIR (Fast Health Interoperability Resources). HL7 FHIR uses modern web technologies and Representational State Transfer APIs, offering a more flexible approach to data exchange [32]. Through Representational State Transfer APIs, different software systems can communicate with each other in real time using HTTP methods. These interactions allow systems to send requests and receive responses, making it possible to retrieve, submit, update, or delete data securely and efficiently [6,44,45].

After setting up the basic technical framework for interoperability (see Figure 1A), the next step involves syntactic and semantic interoperability (see Figure 1B,C). These 2 levels ensure that data are not only structured in a standardized format but also consistently understood across systems [31,32]. In genomics, this is crucial due to the precision required in interpreting variant information. For this reason, the HL7 Clinical Genomics Work Group [46] developed the FHIR Genomics Reporting IG [47]. The HL7 FHIR Genomics Reporting IG defines a set of standardized profiles for key FHIR resources such as ServiceRequest, Specimen, Observations, DiagnosticReport, Task, and DocumentReference (see Figure 2) [45].

Each of these resources uses formats like JSON for data representation and relies on widely adopted coding systems. Examples include Logical Observation Identifiers Names and Codes (LOINC) [48] for laboratory test types, ClinVar [49] for variant classification, the Human Genome Variation Society (HGVS) [50] nomenclature for genetic variant notation, and Systematized Nomenclature of Medicine Clinical Terms [51] or HUGO Gene Nomenclature Committee (HGNC) [52] for gene and condition terminology. The combination of structured formats and standardized vocabularies enables consistent interpretation across systems and enhances machine readability of genomic information. In EHR systems that incorporate CDSSs, this structured information may be used to generate timely alerts, treatment recommendations, or risk assessments. Nevertheless, these tools complement clinical expertise and should be used alongside appropriate genomics training, professional judgment, and collaboration with clinical genetics specialists and laboratories [45].

Of course, none of these would be possible without ensuring the protection of patient data (see Figure 1D,E) [6]. In Europe, this is required by the General Data Protection Regulation (GDPR), which sets strict rules for how personal health information is handled [53]. HL7 FHIR helps support GDPR compliance through Substitutable Medical Applications, Reusable Technologies, a framework that adds security and access control features. Substitutable Medical Applications, Reusable Technologies on FHIR uses the OAuth 2.0 framework and ensures that approved apps can access patients’ data only with the appropriate permissions [45]. This helps keep patient information safe and private. It also allows patients to access their own health data, participate in their own care, and make informed health decisions [7,20,21]. The flexible architecture of FHIR makes it ideal for laboratory communications, allowing systems to easily exchange and protect data. It provides a modern alternative to HL7 v2, supporting the integration of complex data types like genomics into EHRs effectively [45].

## Lessons From Past Initiatives: The Electronic Medical Records and Genomics and Clinical Sequencing Evidence-Generating Research Experiences

The HL7 FHIR Genomics Reporting IG was first published in November 2019 [54]. As a relatively recent standard, most published experience comes from pilot implementations and demonstration projects rather than everyday clinical practice [19,55,56]. However, earlier initiatives, including the Electronic Medical Records and Genomics (eMERGE) Network and the Clinical Sequencing Evidence-Generating Research (CSER) program, had already explored the integration of genomic test results into EHRs [20,29,57]. These early efforts revealed a range of implementation challenges, some of which align with the broader issues described earlier in the manuscript:

- Variability in EHR systems and clinical workflows: The eMERGE and CSER initiatives involved multiple institutions, each with its own approach to managing EHR infrastructure and clinical processes. This made it difficult to apply a consistent approach to genomic data integration.
- Reliance on unstructured reporting formats: In many cases, genomic test results were not provided in a structured format. Instead, they were delivered as PDF documents or narrative text. These formats did not include discrete data fields. Consequently, it was difficult to extract or use information in clinical systems. This limited their usefulness for decision support or automated processing.
- Limited EHR support for genomic findings: Most EHRs did not include dedicated fields for storing genetic variants. These methods were often manual and prone to error. At the time, standards like HL7 FHIR Genomics Reporting IG were still under development and did not fully meet real-world needs.
- Underdeveloped decision support tools: Automated decision support relies on structured, computable data. Without it, most systems could not trigger genetic alerts or recommendations. eMERGE and CSER noted this as a major gap.
- Workflow, education, and organizational barriers: Many clinicians lacked sufficient training in genetics. As a result, they were often unsure how to interpret or act on findings. In theory, CDSSs could have helped bridge this gap. However, as discussed above, genomic results were not yet available in structured and standardized formats. Thus, CDSS tools were difficult to implement. This made clinician training essential for applying genomic data in practice.

The eMERGE and CSER initiatives demonstrated both the feasibility and the complexity of incorporating genomic information into clinical systems. Their experience indicates that technical exchange standards must be accompanied by consistent content definitions, clinically appropriate workflows, governance arrangements, and professional support. Building on these lessons, the NeHA, UCY, and CING organized a workshop within the framework of the Xt-EHR and CY-EHDS-2nd projects to investigate the necessary requirements for integrating genomics into EHR systems.

## Toward Practical Genomic Integration: Current Practice, Gen2EHR Workshop, and a Proposed Roadmap

Based on what has been mentioned in the previous sections, we now present the practical perspective of this viewpoint for integrating genomic findings into EHRs. We briefly introduce the European initiatives supporting this work, describe the current genomic service delivery workflow at CING, summarize the main outcomes of the Gen2EHR IG Workshop, and present a practical roadmap for implementation.

### European Context: The Xt-EHR and CY-EHDS-2nd Initiatives

EHDS aims to enable secure and interoperable access to, exchange of, and use of electronic health data across Member States for both health care delivery and research. Achieving this vision requires interoperability at all levels so that health information can be exchanged, interpreted, and reused consistently across different health care systems [12,13,15].

To support the implementation of the EHDS, the European Commission has launched several complementary initiatives [18]. Two of these are the Xt-EHR Joint Action and the CY-EHDS-2nd project, under which the Gen2EHR IG Workshop was organized [16,17].

Within this framework, the Xt-EHR Joint Action supports the primary use of health data by developing the European Electronic Health Record Exchange Format and technical specifications for cross-border exchange of health information [16,58]. In parallel, CY-EHDS-2nd supports the governance and technical infrastructure required for the secondary use of health data in Cyprus, including Health Data Access Bodies and Secure Processing Environments, in compliance with the GDPR [17,53,59,60].

### Current Genomic Reporting Practices: The CING Institutional Case Study

This section presents the genomic testing and reporting workflow at CING as a real-world institutional case study. It illustrates the practical challenges of integrating genomic information into EHRs. Although the workflow reflects current practice at CING, many of the challenges identified are consistent with those reported in the literature and are relevant to other health care organizations [34].

Within this broader European framework, CING operates as a translational research center that combines molecular diagnostics, clinical services, research, and education. Through its participation in the Xt-EHR Joint Action, CING has contributed an operational perspective to discussions concerning the integration of clinically relevant genomic data as a key component of the EHDS. The aim is to leverage genetic information to support more personalized and preventive care for patients across Europe. At the same time, it opens new possibilities for research and the design of targeted therapies.

At CING, genomic testing is part of routine diagnostic services. The process begins with DNA sequencing and continues through multiple stages of bioinformatics processing, variant detection, annotation, interpretation, and clinical reporting (Figure 3). Each stage generates different data types that vary substantially in size, complexity, format, and intended purpose. Some outputs are required for laboratory analysis, whereas others are intended to support clinical decision-making.

**Figure 3.**
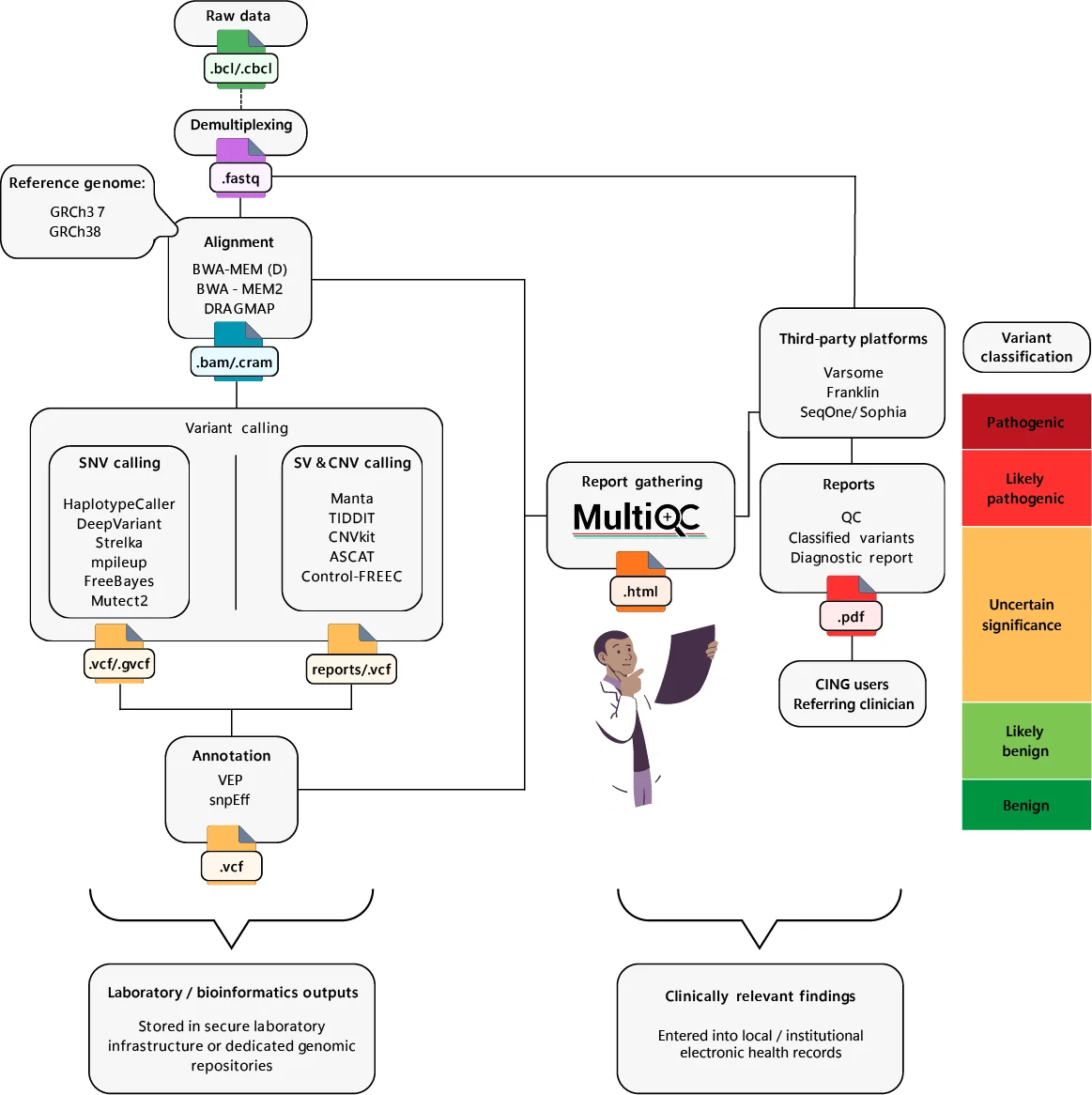
Current genomic diagnostic workflow at CING. The workflow summarizes the laboratory and bioinformatics steps from DNA sequencing to the final clinical report. It also illustrates the different genomic data types generated throughout routine diagnostics. A complete list of the bioinformatics tools used at each stage of the workflow is provided in Multimedia Appendix 1. BAM: Binary Alignment/Map; BWA-MEM: Burrows-Wheeler Aligner-Maximal Exact Matches; CING: Cyprus Institute of Neurology and Genetics; CNV: Copy number variant; CRAM: Compressed Reference-oriented Alignment Map; DRAGMAP: DRAGEN Mapper; GRCh37: Genome Reference Consortium Human Genome build 37; GRCh38: Genome Reference Consortium Human Genome build 38; gVCF: Genomic Variant Call Format; QC: Quality control; SnpEff: Single-nucleotide polymorphism Effect (variant annotation tool name); SNV: Single-nucleotide variant; SV: Structural variant; VCF: Variant Call Format; VEP: Variant Effect Predictor.

Importantly, Figure 3 and Table 1 do not suggest that every file generated during genomic analysis should be incorporated into the EHR. Instead, they illustrate the complexity of the laboratory workflow and the variety of genomic data produced before a clinical diagnosis is reached. Most of these data are laboratory resources rather than clinical information. They include raw sequencing files (FASTQ), alignment files (BAM/CRAM), variant call files, quality-control outputs, and other bioinformatics results. As summarized in Table 1, these data differ considerably in their size, format, and intended purpose. They also present different challenges for integration into EHR systems. Most should remain within specialized laboratory infrastructures or dedicated genomic repositories. By contrast, only clinically meaningful information should be represented in the EHR. This includes validated variants, standardized phenotype information, diagnostic interpretations, and clinical recommendations. Whenever appropriate, these structured records should also include links to the underlying laboratory resources.

**Table 1.** Genomic data generated during routine diagnostics and the proposed information boundary for electronic health records integration^a^.

Data type	File format	WES^b^ file size	WGS^c^ file size	Standardized format	Challenges for integrating into EHRs^d^	Recommended location
Raw base calls	FASTQ	~5 GB	~80 GB	Yes	Raw read format cannot be interpreted, but it is necessary for realignments on future/updated reference genomes	Laboratory repository
Aligned reads	BAM/CRAM	~5 GB	~100 GB	Yes	Large files that require specific software pipelines to be accessed for visualization or other uses	Laboratory repository
Variant callset	VCF^e^	~5 MB	~3 GB	Yes	Needs to be annotated to be interpretable. Can be used for targeted queries for known variants	Laboratory repository/reference database
Annotated variants	VCF/TSV/TXT/SQL	<2 ΜΒ	<2 ΜΒ	No	Numerous annotation sources, including population frequency databases, effect and pathogenicity predictors, and gene model/transcript-specific annotations, etc. Need to shortlist what is important and necessary	Structured repository linked to EHR
Classified variants	TSV/TXT/PDF/XLSX/SQL	<2 ΜΒ	<2 ΜΒ	No	ACMG^f^/AMP^g^ classification cannot be fully automatic due to the ACMG 2015 definitions of specific criteria. In addition, there is a dependency on heavy annotation sources and ambiguity in some criteria definitions.	Structured EHR
Diagnostic reports	.PDF or other formats	<2 ΜΒ	<2 ΜΒ	No	Shortlisted variants that are pathogenic, likely pathogenic, and, in some cases, of uncertain significance, but with relevance to the referred disease/phenotype of interest. Limiting it to secondary uses or primary uses for other conditions	EHR (structured and human-readable)

a Overview of the genomic data types generated throughout the diagnostic workflow. The table highlights their file formats, approximate file sizes, standardization status, challenges for EHR integration, and recommended storage location.

b WES: whole exome sequencing.

c WGS: whole genome sequencing.

d EHR: electronic health record.

e VCF: Variant Call Format.

f ACMG: American College of Medical Genetics and Genomics.

g AMP: Association for Molecular Pathology.

Although the workflow is specific to CING, the challenges it highlights are not unique. Similar barriers have been reported across laboratories and health care organizations working to integrate genomic information into EHRs [20,29,57]. These shared challenges provided the foundation for the Gen2EHR IG Workshop, which brought together multidisciplinary experts to define common requirements and develop practical implementation recommendations.

### Gen2EHR Workshop: Practical Perspectives on Genomic Integration

The recommendations presented in this section reflect our perspective on the practical integration of genomic information into EHRs. They are based on the evidence discussed throughout this viewpoint and were further informed by the Gen2EHR IG Workshop. They should therefore be interpreted as expert-informed implementation recommendations rather than a formal consensus statement.

#### Practical Insights From the Workshop

The workshop was held on September 29‐30, 2025, and was co-organized by CING, NeHA, and UCY. Approximately 50 participants attended the first day. These included invited contributors from the Xt-EHR Joint Action and the CY-EHDS-2nd project representing several European countries, including Czechia, Croatia, Latvia, Hungary, Sweden, and Cyprus. Participants were invited based on their expertise and involvement in clinical genetics, laboratory diagnostics, bioinformatics, health informatics, eHealth, and the implementation activities of the Xt-EHR Joint Action and the CY-EHDS-2nd project. Representatives from ministries of health, national eHealth authorities, health care organizations, academia, and research institutions also participated. The workshop further benefited from invited expertise from the international HL7 FHIR community, providing practical perspectives on FHIR implementation and interoperability.

A predefined agenda guided the discussions (Multimedia Appendix 1). The first day included open scientific sessions covering the complete genomic workflow, from clinical practice and laboratory diagnostics to bioinformatics, interoperability, HL7 FHIR implementation, and clinical decision support. These sessions established the context for the second day, which consisted of an internal working session attended by 10 participants from the Cypriot organizers. The discussions focused on implementation requirements and the development of practical recommendations. They were documented through meeting minutes and draft IG, which were subsequently reviewed by the Xt-EHR consortium partners and refined based on their feedback. No formal voting or consensus procedure was used. Where different perspectives emerged, these were discussed during the workshop and subsequently refined through iterative feedback from the Xt-EHR consortium partners. The resulting guidance is reflected in the published Xt-EHR workshop materials (Multimedia Appendix 2).

Beyond the formal discussions, participants also shared practical implementation experience from their own organizations, including the genomic testing and reporting workflow currently used at CING. These discussions highlighted recurring challenges, particularly the limited interoperability of static genomic reports and the difficulty of searching, updating, and reusing genomic information within EHRs. These practical insights helped shape the implementation recommendations presented in the following section.

#### Key Recommendations for Genomic Integration

Building on the published literature, relevant technical standards, and the practical insights gained from the Gen2EHR Workshop, we recommend adopting a common set of interoperability standards and terminologies for representing genomic information within EHRs. Existing eHealth Network guidelines for exchanging conventional laboratory results provide a useful foundation but should be extended to address the specific requirements of genomic data [61].

To support this approach, Human Phenotype Ontology (HPO) should be used to describe phenotypic features. Combined with reference databases such as OMIM (Online Mendelian Inheritance in Man) and Orphanet, HPO provides a comprehensive framework for describing gene-associated conditions [62-64]. This approach allows each genomic variant stored in the EHR to be linked to structured descriptions of a patient’s symptoms and to standard identifiers for related diseases.

In addition, genomic variants should be described using HGVS nomenclature. HGVS is the international standard for describing DNA- and protein-level changes. Each reported variant should also include the associated gene symbol using HGNC, together with precise genomic coordinates to ensure unambiguous identification. Their clinical significance should be classified according to the American College of Medical Genetics and Genomics (ACMG)/Association for Molecular Pathology and Association for Clinical Genomic Science guidelines, using defined categories such as pathogenic, likely pathogenic, and VUS [65-69]. Recording this information in a structured format allows EHR systems not only to distinguish between confirmed and uncertain findings but also to support the longitudinal reinterpretation of genomic variants. As new evidence emerges and similar cases are identified, variants previously classified as VUS may be reclassified, improving diagnostic yield and enabling more accurate clinical management [40,41,70].

The same standardized approach should be extended to genetic test identifiers and pharmacogenomic information. LOINC codes should be used to identify genetic tests consistently across laboratories [71]. In addition, standardized vocabularies for medications and gene-drug interactions should be incorporated to support pharmacogenomic clinical decision-making. This would allow automated alerts that inform clinicians about drug efficacy, dosage recommendations, and potential side effects based on a patient’s genetic profile.

Finally, genomic information should follow a standardized data model, like the role of Digital Imaging and Communications in Medicine in radiology. Digital Imaging and Communications in Medicine enables the consistent representation and exchange of medical imaging across health care systems [72]. Similarly, widely adopted standards, including HPO [64], HGVS [68], OMIM/Orphanet [62,63], ClinVar [49], ACMG [69], and LOINC [71], provide the foundation for representing genomic results as structured, computable, and shareable data rather than static documents. This approach will improve consistency, support clinical decision-making, facilitate variant reinterpretation, and enable the scalable integration of genomics into routine health care.

#### Proposed Roadmap: From Local Implementation to European Alignment

Building on the recommendations presented above, we propose a practical roadmap for implementing genomic information within EHRs (Figure 4). The roadmap follows a stepwise approach, beginning with national implementation and progressing toward broader European alignment. Although informed by the Xt-EHR Joint Action and the CY-EHDS-2nd project, it should be considered a practical implementation strategy rather than an established European policy.

**Figure 4.**
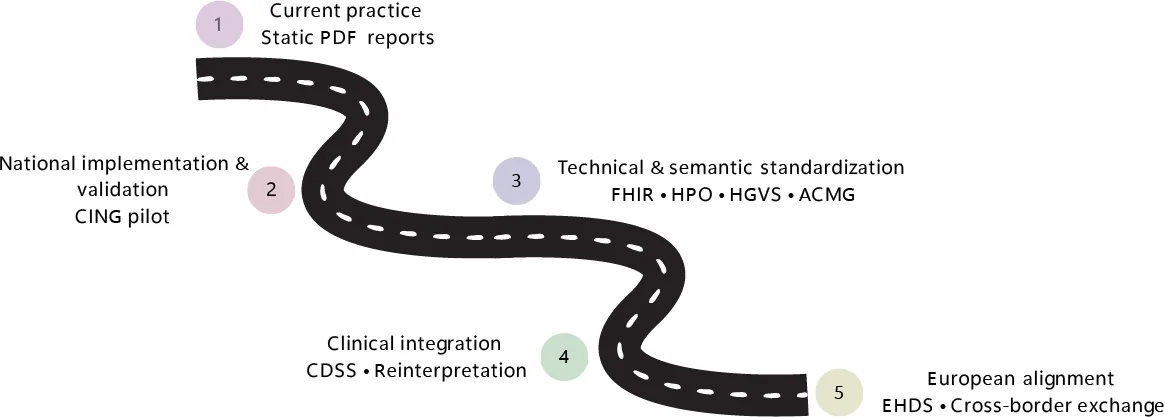
Proposed roadmap for integrating clinically relevant genomic information into electronic health records (EHRs). The roadmap illustrates the proposed stepwise transition from current static PDF-based genomic reports toward interoperable and clinically actionable genomic data within EHR systems. The implementation pathway includes national validation, technical and semantic standardization, clinical integration, and alignment with the European Health Data Space (EHDS) to enable secure cross-border genomic data exchange. ACMG: American College of Medical Genetics and Genomics; CDSS: clinical decision support system; CING: Cyprus Institute of Neurology and Genetics; FHIR: Fast Health Interoperability Resources; HGVS: Human Genome Variation Society; HPO: Human Phenotype Ontology.

The first step focuses on national implementation. We propose that the recommendations presented in this viewpoint are first implemented and evaluated in Cyprus. This will identify practical challenges and assess the proposed specifications in a real clinical setting. The experience gained can then be used to refine the approach before broader European adoption.

The second step focuses on technical implementation. We recommend using the HL7 FHIR Genomics Reporting IG as the foundation for representing and exchanging genomic information [47,56,73]. A pilot project should map the complete diagnostic workflow, from sample collection to the final clinical report, using FHIR resources. This will evaluate whether the required genomic and clinical information can be represented in a structured and interoperable format.

During the transition period, laboratories may continue to provide both a human-readable PDF report and a structured FHIR message. This allows existing clinical workflows to continue while structured genomic reporting is gradually introduced into EHRs. As interoperability standards mature, the structured FHIR message should become the primary format for electronic exchange.

Once validated nationally, the proposed framework can be shared with the Existing eHealth Network and other relevant European initiatives. The goal is to contribute practical experience that supports future harmonization within the EHDS. At the same time, national adaptations will remain necessary to reflect differences in health care systems while maintaining alignment with common interoperability standards.

Overall, this roadmap supports the gradual implementation of genomic information rather than a single large-scale deployment. Experience from national pilot projects can guide future refinements before wider adoption. This approach will reduce the risk of fragmented solutions, improve interoperability, and support the secure exchange and reuse of genomic information across European health care systems.

#### Illustrating the Gap: From Static Reports to Actionable Genomic Information

The need for this transition was demonstrated during the Gen2EHR IG Workshop using a de-identified clinical case from routine diagnostic practice at CING. The case highlighted a key limitation of current genomic reporting. Established standards and coding systems are already used to describe clinically relevant findings, but this information remains embedded in static reports rather than represented as structured, interoperable data.

The case involved an individual with a complex neurodevelopmental disorder who underwent whole exome sequencing. Two pathogenic variants in the ADGRG1 gene were identified and considered consistent with the patient’s condition. The report described the variants using HGVS [68] nomenclature and classified them according to the ACMG [69] guidelines. The phenotype was documented using HPO [64] terms, the diagnosis was linked to an OMIM [62] identifier, and recommendations for family testing were included. Despite the use of these established standards and terminologies, the final report was provided as a PDF and stored in the EHR as a static attachment. The individual data elements were therefore not available in a structured or FHIR-based format.

As this example originated from routine clinical practice, its use was limited to the de-identified information needed to demonstrate the reporting workflow. Written informed consent for genetic testing and for the anonymized use of clinical and genetic information for research purposes was obtained from the patient or, when necessary, from a parent or legal guardian, in accordance with CING procedures. No additional testing, intervention, or clinical data collection was undertaken for the workshop or this viewpoint.

The case therefore illustrates a practical distinction between standardized reporting and structured data integration. Current reporting practices allow clinically relevant information to be communicated using established standards, but the PDF format limits its direct computational use. If new evidence changed the interpretation of a variant or identified new therapeutic implications, clinicians would need to retrieve the original report and review the updated evidence manually. In addition, only the clinically relevant variants identified at the time of analysis are documented in the report, limiting the future reuse of genomic information.

Representing these elements using standardized terminologies together with the HL7 FHIR Genomics Reporting IG could bridge this gap by transforming the findings into structured, computable data. This could support genotype-based clinical decision support, facilitate future variant reinterpretation, and improve the exchange of genomic information between health care systems. Genomic results could therefore become an active part of the patient’s longitudinal health record rather than remain a static laboratory report.

## Discussion

### Practical Considerations for Genomic Integration into EHRs

Integrating genomics into EHRs is no longer limited by the ability to generate genomic data. The main challenge is representing, interpreting, updating, and reusing clinically relevant findings in routine care [6,7,20]. Early initiatives such as eMERGE and CSER showed that genomic information can be incorporated into clinical systems, but they also highlighted fragmented workflows, limited interoperability, and continued reliance on static reports [20,29,74,75]. As a result, genomic information remains difficult to integrate with phenotypes, diagnoses, medications, and longitudinal care [21]. These experiences demonstrate that storing genomic reports in EHRs is not enough. Clinically relevant findings must be represented as structured, interoperable data that can support ongoing patient care [2].

This viewpoint focuses on the practical steps needed to achieve this goal. We propose a roadmap that begins with national implementation and aligns with broader European initiatives such as Xt-EHR and CY-EHDS-2nd. It is based on shared interoperability standards, structured reporting, and the HL7 FHIR Genomics Reporting IG. Rather than a fixed model, it is intended as a practical framework that can be evaluated and refined through real-world implementation.

A key message from both the literature and the Gen2EHR Workshop is that EHRs should capture clinically relevant findings rather than raw sequencing data. Files such as FASTQ and BAM are better maintained within secure laboratory infrastructures or dedicated genomic repositories. EHRs should instead contain standardized variants, clinical interpretation, and associated phenotypic information. Standards including the HL7 FHIR Genomics Reporting IG, HGVS, HPO, LOINC, HGNC, and ACMG/Association for Molecular Pathology provide the foundation for consistent genomic data exchange. As genomic knowledge evolves, structured systems must also support versioning and variant reinterpretation.

However, technical standards alone are not sufficient. Successful implementation also depends on clear clinical workflows, appropriate governance, and access to genetics expertise. Clinicians need reports that are easy to interpret, while patients should understand how their genomic data are stored, used, and updated. Building trust among both groups will be essential for routine implementation.

A gradual transition from conventional reports to structured genomic information is likely to be more practical than replacing existing workflows at once. During this period, PDF reports and structured FHIR messages may coexist while health care systems progressively adopt interoperable solutions. Pilot implementations should evaluate technical performance together with usability, workflow integration, clinical decision-making, and clinical decision support in routine care.

Finally, the roadmap presented in this viewpoint should be regarded as a proposed implementation strategy rather than a validated model. It reflects our interpretation of the literature together with the practical perspectives shared during the Gen2EHR Workshop. Future pilot studies will determine how well this approach performs in different health care settings and help refine it before broader adoption across Europe.

### Limitations and Transferability

This viewpoint is informed by published literature, technical standards, the operational experience of CING, and recommendations developed through the Gen2EHR Workshop and reviewed by the Xt-EHR consortium. It does not present a systematic review or findings from an implemented and clinically evaluated genomic EHR system. In addition, the CING workflow reflects the experience of a single highly specialized institution within the Cypriot health care system. Although the proposed use of internationally adopted interoperability standards and genomic terminologies is intended to support broader transferability, the roadmap has not yet been evaluated across different health care settings. It should therefore be interpreted as a practical implementation strategy rather than a validated or universally applicable model.

The proposed framework also does not define the governance processes required for implementation. Issues such as variant reinterpretation, communication of updated findings, patient recontact, and the return of clinically relevant information require clearly defined roles and responsibilities. These processes are influenced by national legislation and institutional policies and will need to be addressed locally as genomic information is integrated into routine care.

The same principles apply to the ethical use of clinical and genomic information. For the clinical example presented in *Illustrating the Gap: From Static Reports to Actionable Genomic Information*, consent, confidentiality, and deidentification were handled in accordance with CING procedures, as described above. Ethics committee approval was not sought because the case was not investigated as part of a research study and only deidentified information from routine clinical practice was used. This approach was considered within the national framework for the ethics review of biomedical research in Cyprus [76].

### Conclusion

Genomic information has the potential to become an integral part of routine health care, but achieving this requires more than storing laboratory reports in EHRs. Structured representation, common interoperability standards, and practical implementation strategies are essential to make genomic findings reusable, interpretable, and clinically actionable. Through the integration of current evidence, practical experience from CING, and insights from the Gen2EHR Workshop, this viewpoint proposes a roadmap to support this transition. Although further evaluation in real-world settings is needed, we hope these recommendations will contribute to ongoing national and European efforts toward interoperable genomic integration within the EHDS.

## Supplementary material

10.2196/99130 Multimedia Appendix 1 Overview of bioinformatics tools used throughout the Cyprus Institute of Neurology and Genetics genomic workflow.

10.2196/99130 Multimedia Appendix 2 Supporting materials from the Gen2EHR Workshop, including the workshop invitation, agenda, workshop outcomes, and press release.

## Abbreviations

ACMG: American College of Medical Genetics and Genomics
CDSS: clinical decision support system
CING: Cyprus Institute of Neurology and Genetics
CSER: Clinical Sequencing Evidence-Generating Research
CY-EHDS-2nd: European Health Data Space for Secondary Use @CY
EHDS: European Health Data Space
EHR: electronic health record
eMERGE: Electronic Medical Records and Genomics
FHIR: Fast Health Interoperability Resources
GDPR: General Data Protection Regulation
HGNC: HUGO Gene Nomenclature Committee
HGVS: Human Genome Variation Society
HL7 v2: Health Level 7 version 2
HPO: Human Phenotype Ontology
IG: Implementation Guide
LOINC: Logical Observation Identifiers Names and Codes
NeHA: National eHealth Authority of Cyprus
OMIM: Online Mendelian Inheritance in Man
UCY: University of Cyprus
VUS: variant of uncertain significance
WGS: whole genome sequencing
Xt-EHR: Extended EHR@EU Data Space
