# Supplementary material for: Making Genomic Data Actionable in Electronic Health Records: Practical Viewpoint on Standards, Governance, and Implementation
Source: JMIR Med Inform. 2026 Oct 21;14:e99130. doi: 10.2196/99130 (PMC13686338; doi:10.2196/99130)
Supplement: Supplementary file 1 [file medinform-v14-e99130-s002.pdf]

Co-funded by the European Union

My health @ EU
eHealth Digital Service Infrastructure
A service provided by the European Union

Extended EHR@EU Data Space for Primary Use - Xt-EHR

Proposal number: 101128085

Gen2EHR Workshop2 – Sept 2025

Integrating Genomic Data into EHR

Co-Funded by the European Union. Views and opinions expressed are however those of the author(s) only and do not necessarily reflect those of the European Union or HaDEA. Neither the European Union nor the granting authority can be held responsible for them.

Author(s)

Name	Surname	Organisation
George	Spyrou	Cyprus Institute of Neurology and Genetics
Theodoros	Kyprianou	National eHealth Authority
Andreas	Menikou	National eHealth Authority
Athina	Theodosiou	Cyprus Institute of Neurology and Genetics
Styliana	Menelaou	Cyprus Institute of Neurology and Genetics

Reviewed by

Name	Surname	Organisation
Christos N.	Schizas	National eHealth Authority
Costas	Pattichis	University of Cyprus
Maria	Papaioannou	University of Cyprus
Andreas	Neocleous	National eHealth Authority
Eirini	Schiza	University of Cyprus
Efi	Evangelou	National eHealth Authority
Savvas	Aristotelous	National eHealth Authority

Abbreviations

CING	Cyprus Institute of Neurology and Genetics
NeHA	National eHealth Authority
EHR	Electronic Health Record
EHDS	Electronic Health Data Space
GDPR	General Data Protection Regulation

Participants

Name	Surname	Organisation
Christos N.	Schizas	National eHealth Authority
George	Spyrou	Cyprus Institute of Neurology and Genetics
Costas	Pattichis	University of Cyprus
Theodoros	Kyprianou	National eHealth Authority
Kyproula	Christodoulou	Cyprus Institute of Neurology and Genetics
Carolina	Sismani	Cyprus Institute of Neurology and Genetics
Menelaos	Pipis	Cyprus Institute of Neurology and Genetics
Andreas	Menikou	National eHealth Authority
Anastasis	Oulas	Cyprus Institute of Neurology and Genetics
Athina	Theodosiou	Cyprus Institute of Neurology and Genetics
Styliana	Menelaou	Cyprus Institute of Neurology and Genetics
Maria	Papaioannou	University of Cyprus
Andreas	Neocleous	National eHealth Authority

Eirini	Schiza	University of Cyprus
Ward	Weistra	Firely
Efi	Evangelou	National eHealth Authority
Savvas	Aristotelous	National eHealth Authority
Erietta	Ioulianou	National eHealth Authority
Marina Ellie	Afami	National eHealth Authority
Nicoletta	Prentza	Univeristy of Cyprus
Anna	Bourouliti	CYENS Centre of Excellence
Eugenia	Bratu	NIPH
Margarida	Carradinha	SPMS
Haralampos	Karanikas	IDIKA SA
Ioannis	Rodosthenous	Univeristy of Cyprus
Cátia	Pinto	SPMS

EXECUTIVE SUMMARY

Summary of the discussions in the closed sessions of the Workshop

Prototype Specification for a Genomics Report in an EHR System

The Gen2EHR Workshop, co-organized by the Cyprus Institute of Neurology and Genetics (CING), the University of Cyprus (UCY), and the National eHealth Authority (NeHA), was held under the EU-funded Xt-EHR Joint Action and the EHDS2nd project. Bringing together clinicians, geneticists, bioinformaticians, policy experts, and technical partners from across Europe, the workshop aimed to define a practical roadmap for integrating genomic data into Electronic Health Records (EHRs). This initiative supports the objectives of the European Health Data Space (EHDS), promoting secure and interoperable health data use for both clinical and research purposes.

Key discussions centered on aligning clinical, laboratory, and technical perspectives to ensure that genomic information becomes a structured, standardized, and computable part of patient records. Participants emphasized the adoption of international standards such as HPO, HGVS, ACMG, and HL7 FHIR Genomic Reporting for consistent data representation, and the importance of privacy-preserving data sharing through GDPR-compliant anonymization and pseudonymization. The integration of genomics was also linked to the future deployment of AI-driven Clinical Decision Support Systems (CDSS), enabling real-time, personalized care and predictive health insights.

As a major outcome, the workshop agreed to establish a national “Genomics-in-EHR” specification within Xt-EHR Work Package 5, with Cyprus serving as a pilot site for implementation and validation. The resulting Gen2EHR Guidelines will provide a reference model for interoperable and privacy-respecting genomic integration in EHRs, offering a scalable blueprint for other EU Member States as the EHDS framework evolves.

TABLE OF CONTENTS

1 Introduction 6
2 Purpose and Scope 6
3 Stakeholders 6
4 Report Structure 7
5 Data Standards and Interoperability (to be enriched) 7
6 Privacy and Security (to be revisited) 8
7 Implementation Step 8
7.1 Define scope & use cases 8
7.2 Decide on phenotype & disease representation 8
7.3 Decide on variant representation 8
7.4 Map your data model to FHIR resources (minimal viable payload) 8
7.5 Terminologies & value sets 9
7.6 Build the lab → EHR integration flow 9
7.7 Re-analysis, versioning, and audit 9
7.8 Validation, conformance, and rollout 9
8 Future Enhancements 10
9 Appendices 10
9.1 Appendix A: Workshop Invitation 10
9.2 Appendix B: Workshop Agenda 11
9.3 Appendix C: Workshop Outcomes 13
9.4 Appendix D: Press Release 17

1 INTRODUCTION

This document presents a prototype specification for a genomics report used in the Cyprus Institute of Neurology and Genetics (CING), a Centre of Excellence in Genomics and affiliated entity to the National eHealth Authority (NeHA). The report is intended for integration within an Electronic Health Record (EHR) system, following the specifications of Electronic Health Data Space (EHDS) and Xt-EHR. The goal is to ensure that genomic data is presented in a structured, clinically meaningful way, supporting clinical decision-making longitudinally and sustainably, while safeguarding patient privacy and compliance with relevant standards. This work is based on the outcomes of a 2-day workshop that took place in CING, within the context of the Xt-EHR EU-funded project (WP5).

In addition to the core content of this deliverable, a set of supporting materials will be provided in the appendices to ensure transparency, reproducibility, and completeness of the workshop process. These include:

- **Appendix A:** Workshop Invitation
- **Appendix B:** Workshop Agenda
- **Appendix C:** Workshop Outcomes
- **Appendix D:** Press Release

2 PURPOSE AND SCOPE

The genomics report specification is designed to:

- Enable the secure and accurate reporting of genomic test results within an EHR.
- Facilitate interpretation by healthcare professionals in a sustainable way.
- Support interoperability between healthcare systems through standardised data exchange formats and coding systems.
- Ensure patient privacy and data protection in accordance with applicable regulations.

3 STAKEHOLDERS

- Clinicians (e.g., clinical geneticists, GPs, specialists, genetic counsellors)
- Laboratory staff
- Patients and carers
- IT, Bioinformatics, and EHR developers

- Regulatory and compliance officers

4 REPORT STRUCTURE

The genomics report will comprise the following key sections:

1. Patient Information, test general info, patient phenotype as per eHN guidelines – p23-28 (Annex 1)
2. Identified variants (e.g., gene, location, zygosity, effect, variant type)
3. Inheritance (Mendelian)
4. Classification (e.g., pathogenic, likely pathogenic, VUS, benign)
5. Interpretation criteria (reference)
6. Interpretation summary (narrative explanation for clinicians, structure i.e. classification, local and international population databases, in silico tool prediction, gene disease correlation, recurrence risk, conclusion)
7. Clinical Recommendations, suggested follow-up actions and referral to genetic counselling
8. Methodology (sequencing platform, library, bioinformatics pipeline version, reference genome, classification guidelines)
9. Sensitivity of the test
10. Limitations
11. Disclaimer
12. References

5 DATA STANDARDS AND INTEROPERABILITY (TO BE ENRICHED)

- Use of internationally recognised coding systems (e.g., SNOMED CT, LOINC, HGVS nomenclature for variants).
- Structured data representation (e.g., HL7 FHIR Genomics resources) to enable data sharing between systems.
- Support for both human-readable and machine-readable report formats.

6 PRIVACY AND SECURITY (TO BE REVISITED)

- Compliance with GDPR and local data protection regulations.
- Role-based access controls within the EHR system.
- Audit logging of access and modifications.
- Secure storage and encrypted transmission of genomic data.

7 IMPLEMENTATION STEP

This prototype specification outlines the essential elements and considerations for the development of a genomics report within an EHR system. It aims to promote consistency, interoperability, and clinical utility, while ensuring the privacy and security of sensitive genomic information.

7.1 Define scope & use cases

- Start with 1 report type: diagnostic germline WES
- Decide which results are structured (variants, interpretations, PGx phenotypes) vs narrative PDFs (attach as DocumentReference-Annex1).
- Clarify re-analysis policy (when/why to issue amended interpretations).

7.2 Decide on phenotype & disease representation

- Capture phenotype using HPO-coded FHIR Observations; diseases as FHIR Condition with Orphanet / SNOMED / ICD codes.
- Consider use of / mapping to GA4GH Phenopackets

7.3 Decide on variant representation

- Record specific guidelines for example, ACMG/AMP classification (Pathogenic / Likely pathogenic / VUS / Likely benign / Benign) and the reference to evidence criteria that led to it.
- Record your curation pipeline and tooling.

7.4 Map your data model to FHIR resources (minimal viable payload)

For example, each genomic order → report:

- Order/indication: ServiceRequest (+ Condition/ICD/ORPHA).

- Patient & consent: Patient, Consent (data use, re-contact).
- Specimen(s): Specimen (collection, container), link to GenomicStudy.
- Study/process: GenomicStudy (assays, references, QC metrics, regions/genes assessed).
- Results:
  - DiagnosticReport (Genomic Report profile) as the wrapper.
  - One or more variant Observations (SNVs/indels/CNV/SVs) with interpretation components.
- Provenance & packaging: Provenance + a Bundle (type = "collection").

7.5 Terminologies & value sets

Genes/variants: HGNC, RefSeq/Ensembl;

- Phenotypes: HPO.
- Diseases: Orphanet, SNOMED CT, ICD-10/11, OMIM
- Labs/biomarkers: LOINC, units via UCUM.

7.6 Build the lab → EHR integration flow

- Inbound (from LIMS/NGS dataflow): produce a FHIR Bundle containing GenomicStudy, DiagnosticReport, Observations, Specimen.
- Co-attach the signed PDF (full report) as DocumentReference.
- Store on your FHIR server (for example HAPI-FHIR R5), link to patient's chart.

7.7 Re-analysis, versioning, and audit

- Track assay version, pipeline, references in GenomicStudy; use Provenance and DiagnosticReport.status for amendments; keep superseded vs current.

7.8 Validation, conformance, and rollout

- Validate instances and run terminology checks (HPO/LOINC/UCUM).

8 FUTURE ENHANCEMENTS

- Integration of decision support tools based on genomic findings.
- Automated alerts for actionable variants,
- PGx applications

9 APPENDICES

9.1 Appendix A: Workshop Invitation

Gen2EHR Implementation Guidelines Workshop (29–30 September 2025)

“Dear colleagues,

You are cordially invited to the **Gen2EHR Implementation Guidelines Workshop**, scheduled for **29–30 September 2025** at the Amphitheater of the Cyprus Institute of Neurology and Genetics in Nicosia.

The Gen2EHR workshop is being organized and funded by the National eHealth Authority of Cyprus (the Competent Authority for eHealth), and its Affiliated Entities, the Cyprus Institute of Neurology and Genetics, and the University of Cyprus. It will serve as a forum to establish the prototype core design for the data flow from the citizen’s genome to the Electronic Health Record (EHR).

Under the European Health Data Space Regulation which has been put in operation last March, a primary-use EHR will establish effective procedures for the secondary use of health data and will define the requirements for integrating genomic and other related health data into the EHR data flow. In turn, insights from the secondary use of health data will support healthcare providers in offering better services to citizens, policy makers, and research organizations.

The work to be presented is co-funded by the EU through two projects: the ***Extended EHR@EU Data Space for Primary Use (Xt-EHR) Joint Action***, coordinated at the EU level by the National eHealth Authority, and the ***European Health Data Space for Secondary Use @CY (EHDS2nd),*** also coordinated by the National eHealth Authority.

Day 1 (29/9/2025) will feature open sessions; Day 2 (30/9/2025) will include closed working sessions for invited participants, concluding with an open session announcing the workshop outcomes. Please see the attached agenda for details. Your active participation in the workshop and your scientific knowledge will contribute to the success of the initiatives that Cyprus has undertaken for benefiting every European citizen.

Kindly confirm your attendance if you plan to participate in person by using the following registration link:

For remote participation, please follow the link below:

Looking forward to your positive response

Constantinos Pattichis, President - National eHealth Authority of Cyprus

Organizers:

Christos N. Schizas, Coordinator Xt-EHR and EHDS2nd

George Spyrou, Participant Xt-EHR and EHDS2nd

Theodoros Kyprianou, Task leader Xt-EHR and EHDS2nd"

9.2 Appendix B: Workshop Agenda

"Integrating Genomics into the EHR" - Gen2EHR Implementation Guidelines Workshop: 29 - 30 September 2025 at The Cyprus Institute of Neurology and Genetics Nicosia, Cyprus

Day 1: MONDAY 29/09/2025		
**8:30 – 9:00 (CY time)**	**Welcome and registration**	
**9:00 – 9:15 (CY time)**	**(OS) Opening Session:** Welcome Notes	**Co-organizers:**<br>Prof. Christos Schizas<br>Prof. Costas Pattichis<br>Dr. George Spyrou
**9:15 – 9:45 (CY time)**	**(OS) Clinical Perspective: Introduce the EHR based on the EEHRxF format and the EHDS Regulation from the clinical point of view –** clinical routines and workflow.	**Clinicians:**<br>Dr. Theodoros Kyprianou
**9:45 – 10:15 (CY time)**	**(OS) Clinical Perspective: Integrating Genomics into EHR for Personalized Medicine** – How genomic data enhances clinical decision-making and patient outcomes.	**Clinicians:**<br>Dr. Menelaos Pipis
**10:15 – 10:30 (CY time)**	**(OS) Lab Workflow I: From Sample to Report** – How lab experts generate genomic diagnostic reports to guide therapy decisions	**Lab Experts:**<br>Dr. Kyproula Christodoulou
**10:30 – 10:45 (CY time)**	**Coffee Break & Networking**	
**10:45 – 11:00 (CY time)**	**(OS) Lab Workflow II: From Sample to Report** – How lab experts generate genomic diagnostic reports to guide therapy decisions	**Lab Experts:**<br>Dr. Sismani Carolina
**11:00 – 11:15 (CY time)**	**(OS) Clinician's Interpretation: Using Genomic Reports for Patient Care** – How clinicians analyze, interpret, and apply lab-generated reports in treatment plans	**Clinicians:**<br>Dr. Menelaos Pipis

**11:15 – 11:30 (CY time)**	**(OS) Challenges in Genomics Data Integration:** What Should Be Included in EHR?	**Bioinformatics Expert:** MSc, Styliana Menelaou
**11:30 – 12:00 (CY time)**	**(OS) FHIR Data Models:** how to gear the FHIR specification towards the needs of your use case, using genomics as an example.	**Ward Weistra,** Product Manager Simplifier/Content Lead FHIR DevDay/Member of the FHIR Management Group
**12:00 – 12:15 (CY time)**	**(OS) Solutions in Genomics Data Integration –** HL7 FHIR Genomics Reporting Implementation Guide	**Medical Informatics Expert:** Dr. Maria Papaioannou
**12:15 – 12:45 (CY time)**	**(OS) Unlocking the Power of Genomic Data for Research & Personalized Medicine: Advancing Clinical Decision Support Systems (CDSS)** – Exploring the secondary use of patient genomic data in clinical research, drug discovery, and AI-driven precision medicine with a focus on enhancing CDSS for improved patient outcomes.	**Clinicians:** Dr. Theodoros Kyprianou & Dr. Andreas Menikou
**12:45 - 13:15 (CY time)**	**Questions - Discussion**	
**13:15 – 14:00**	🍽️ **Lunch Break**	
**14:00– 14:15**	**(OS) State-of-the-art Genomics Data processing –** Data types, Pipelines, Computational Capacity Requirements, GPU-acceleration	**Bioinformatics Experts:** Dr. Marios Tomazou
**14:15 – 14:30**	**(OS) Variant Interpretation and Reporting -** Genomic data filtering, classification, validation, and reporting. Challenges and Clinical Implications	**Bioinformatics Expert:** Dr. Athina Theodosiou
**14:30 – 15:00**	**(OS) Presentation of the Xt-EHR and EHDS2nd projects - Understanding the Existing prototype of EHR**	**Project Managers:** Dr. Andreas Neocleous Dr. Maria Papaioannou
**15:00-15:30**	**Discussion – Questions**	
**Day 2: TUESDAY 30/09/2025**		
**8:30 – 9:00**	**Welcome and registration**	
**9:00 – 11:00**	**(CS) Working Session 1:** Related scientific articles in preparation.	**Session Chairs:** Dr. Eirini Schiza Styliana Menelaou
**11:00 – 13:00**	**(CS) Working Session 2:** Define the requirements/specifications regarding the integration of Genomic Data into the EHR Data Flow, according to the overall Xt-EHR	**Session Chairs:** Dr.Marios Tomazou Dr. Andreas Menoikou

	workflow.	
**13:00 – 14:00**	🍽 **Lunch Break**	
**14:00 – 15:00**	**(CS) Working Session 3:** Summarize the solutions/specifications/recommendations to be announced to the closing event.	**Session Chair:** Dr. George Spyrou
**15:00 – 16:00**	**(CS) Presentation of the Working Sessions outcome -** Round Table Discussion and Refinements on the Workshop’s outcome	**Session Chair:** Prof. Costas Pattichis
**16:00-16:45**	**(OS)** Announcement of updates on the design of the prototype core for the flow of patient genome data into the EHR, supporting primary use and enabling well - designed secondary use of data. Challenges, Future Work. Closing Remarks.	Prof. Christos N. Schizas<br>Prof. Costas Pattichis<br>Dr. Theodoros Kyprianou<br>Dr. George Spyrou
**16:45-17:00**	**(OS) Closing Remarks**	

* Sessions:

- Closed Designing/Working Sessions (CS) with only invited participants from the 3 Organizations (National e-Health Authority of Cyprus (CA), The Cyprus Institute of Neurology and Genetics (AE), University of Cyprus (AE))
- Open Sessions for public disseminations (OS)

9.3 Appendix C: Workshop Outcomes

EXECUTIVE SUMMARY

Summary of the discussions in the closed sessions of the Workshop

Prototype Specification for a Genomics Report in an EHR System

The Gen2EHR Workshop, co-organized by the Cyprus Institute of Neurology and Genetics (CING), the University of Cyprus (UCY), and the National eHealth Authority (NeHA), was held under the EU-funded Xt-EHR Joint Action and the EHDS2nd project.

A central outcome of the workshop was the recognition that genomic data must transition from static, document-based reports to structured, machine-readable formats that can be integrated into EHR systems and support clinical decision-making. This requires the systematic adoption of internationally recognized standards and terminologies, including HPO for phenotype representation, HGVS for variant description, ACMG/AMP guidelines for clinical classification, and LOINC for laboratory tests.

Participants also emphasized the importance of linking genomic findings with detailed and standardized phenotypic data, as well as enabling continuous updates such as variant reclassification and re-analysis. These capabilities are essential for maintaining clinically relevant and up-to-date patient records.

The workshop further defined a clear implementation pathway, starting with the development of national "genomics-in-EHR" guidelines within the Xt-EHR framework, followed by pilot implementation in Cyprus and gradual scaling to EU-level adoption. This approach aligns with the objectives of the European Health Data Space, supporting interoperability, cross-border data exchange, and the advancement of personalized medicine.

Overall, the workshop established a shared vision for the development of dynamic, interoperable, and clinically actionable EHR systems, capable of integrating genomic data into routine healthcare practice

1 TECHNICAL STANDARDS FOR GENOMIC INTEGRATION INTO EHR SYSTEMS

The Gen2EHR Implementation Guidelines Workshop (Sept 29–30, 2025) brought together clinicians, geneticists, bioinformaticians, and policy experts to define the key directions for integrating genomic information into EHR systems. Linking genomics with EHRs is a major step forward for personalized medicine (Abul-Husn & Kenny, 2019). However, existing EHR infrastructures are not yet designed to manage the scale and complexity of genomic data.

During the workshop, two of the participating doctors emphasized the need for EHRs to evolve into comprehensive "records of health" rather than just "records of disease". In other words, they should support prevention and well-being in addition to treatment. To achieve this, the data must be accurate, structured, and standardized. This also ensures that clinical decision support systems can function effectively and provide real assistance to clinicians. When information is recorded as free text, it is hard to interpret genomic findings reliably or use the data for advanced analyses.

Furthermore, participants emphasized that genomic data should be an integral part of the EHR, closely linked to detailed and standardized descriptions of each patient's phenotype and symptoms to support accurate interpretation and variant correlation.

Overall, the workshop underscored that effective genomic integration requires interoperability, structured data, and a shift toward more intelligent, health-centered EHR systems.

2 WORKSHOP INSIGHTS FROM LABORATORY PERSPECTIVES:

To better understand how challenging this integration is, the workshop included valuable insights from laboratory experts. The heads of the Molecular Genetics and Cytogenetics departments at CING shared their experiences, presenting the current practices in genomic reporting and highlighting the main obstacles laboratories face. They explained that NGS reports are generated after variant filtering and expert review, and are typically delivered as 1–2-page PDF documents. These reports follow established standards such as HGVS (https://hgvs-nomenclature.org/stable/) for variant notation, gene names, zygosity, inheritance patterns, and ACMG classification for clinical interpretation, and each concludes with a short diagnostic summary. (Hart et al., 2024; Richards et al., 2015)

However, such static reports are difficult to integrate into EHR systems or to use effectively in clinical decision support, since they cannot be easily searched, linked, or updated. The speakers emphasized that

while it is not feasible to store raw genomic data, the future lies in structured data formats connected to dynamic and continuously updated knowledge resources. They also highlighted that accurate and detailed phenotypic descriptions are critical for correct variant interpretation. Laboratories now request Human Phenotype Ontology (HPO) terms with each NGS referral, as standardized phenotype data enable more precise analyses and reduce the risk of misdiagnosis. (Robinson et al., 2008) Overall, the laboratory perspective underscored that effective genomic integration requires both structured, interoperable data formats and consistent, high-quality clinical information that together can transform static reports into actionable knowledge within EHR systems.

Building on these points, participants emphasized that establishing harmonized national guidelines is only the first step toward a broader, collaborative framework for genomic data management. To ensure consistency and long-term usability, EHR systems must be designed to support continuous updates, version control, and interoperability between laboratories, hospitals, and research databases. This will allow variant reclassifications and newly discovered gene–disease associations to be reflected automatically in patient records, keeping clinical information current and reliable. Ultimately, the goal is to move from fragmented, document-based reporting toward a dynamic ecosystem where genomic and clinical data coexist seamlessly, enabling more accurate diagnoses, better treatment decisions, and continuous learning within healthcare systems.

3 STANDARDS FOR GENOMIC DATA: KEY PROPOSALS FROM GEN2EHR:

A key outcome of the workshop was a shared agreement on the main technical standards and terminologies needed to represent genomic information within EHRs. Participants acknowledged the importance of the existing eHealth Network (eHN) guidelines for laboratory result exchange as a starting point but noted that these frameworks, designed for conventional laboratory tests, must be expanded to address the specific requirements of genomics. (Laboratory Results Guidelines, Release 1.1, Jun 2023, n.d.)

To achieve this, the workshop proposed the adoption of widely used ontologies and nomenclatures that can ensure data consistency across systems. For describing phenotypes and diseases, the use of Human Phenotype Ontology (HPO) terms was strongly recommended, together with reference databases such as OMIM (Online Mendelian Inheritance in Man) and Orphanet for coding specific genetic conditions or syndromes. (Hamosh et al., 2005; Polak & Cavé, 2007; Robinson et al., 2008) This approach would allow each genomic variant recorded in the EHR to be directly linked to structured descriptions of the patient's clinical features (for example, an HPO term for "peripheral neuropathy") and to standard identifiers for the relevant disorder.

For genetic variants, the HGVS nomenclature should be used to describe DNA and protein-level changes, as it remains the international standard for variant reporting. Each reported variant should also include the associated gene symbol (using standardized identifiers such as HGNC) and precise genomic coordinates to ensure accurate identification. The classification of clinical significance should follow the ACMG/AMP guidelines, which define categories such as pathogenic, likely pathogenic, and variant of uncertain significance (VUS). Recording this information in a structured format would allow EHR systems to distinguish between confirmed and uncertain findings, supporting more reliable clinical decision support. (Hart et al., 2024; Richards et al., 2015)

In addition, participants recommended extending these standards beyond variant representation to include the full clinical and pharmacogenomic context of genomic data. The use of LOINC codes was proposed to identify genetic tests consistently across laboratories, while controlled vocabularies for medications and gene–drug interactions would enable the development of pharmacogenomic alerts directly within clinical workflows. This would allow clinicians to receive automated guidance on drug efficacy or potential adverse reactions based on a patient's genetic profile. The overall consensus was that genomics should follow the example of other well-standardized medical domains, such as radiology, where the DICOM model ensures interoperability and consistent interpretation across institutions. By encoding genomic data with widely recognized standards such as HPO, HGVS, OMIM/Orphanet, ClinVar (https://www.ncbi.nlm.nih.gov/clinvar/), ACMG, and LOINC, and organizing results into structured, shareable data fields, EHR systems can move from static document storage to dynamic, interpretable datasets. This standardized and modular data model will be essential for ensuring data consistency, supporting clinical decision-making, and enabling the safe, scalable integration of genomics into everyday healthcare practice. (Bidgood et al., 1997; Hamosh et al., 2005; Hart et al., 2024; Polak & Cavé, 2007; Richards et al., 2015; Robinson et al., 2008; Yeh et al., 2021)

4 FROM NATIONAL GUIDELINES TO EU-WIDE ADOPTION:

The workshop also outlined a clear plan for implementing these standards. The first step is to formalize genomic data standards at the national level, as part of the ongoing Xt-EHR project. Based on the workshop discussions, a draft "genomics-in-EHR" specification is now being developed to define exactly which genomic data should be included in electronic health records and how this information should be structured. Once reviewed and refined nationally, these guidelines are expected to be proposed to the eHealth Network (eHN) and other European bodies to help shape the future implementation of the European Health Data Space.

This bottom-up approach, starting with a national pilot and gradually scaling to the EU level, was seen as a practical and achievable path toward alignment and broad adoption. Cyprus, already active in cross-border eHealth collaboration, will act as a test case to validate and refine the model before it is expanded across Europe. The timing is particularly important, as EU policy envisions that by 2031, all member states will need to support cross-border genomic data exchange under the European Health Data Space framework. Establishing a strong national template now will not only streamline this transition but also position Cyprus as a key contributor to shaping a unified European model for genomic integration.

Once the content and policy standards are finalized, the next focus will be on their technical implementation. Workshop participants agreed that integration should rely on modern interoperability frameworks, particularly the HL7 FHIR Genomics Reporting Implementation Guide (IG). However, this can only happen after the core data standards are clearly defined. In other words, it is essential to decide what data should be included before determining how to exchange it. The FHIR Genomics IG provides a strong foundation for representing genomic findings. It includes structured profiles such as Observation for variants and DiagnosticReport for overall interpretation. Still, these profiles must be further refined and adapted to terminology standards. For example, variant descriptions should follow HGVS notation, while phenotype information should use HPO codes. This alignment will ensure consistency, interoperability, and accurate interpretation across systems. (Hart et al., 2024; Robinson et al., 2008)

The implementation plan includes a pilot that will map a real diagnostic workflow, from sample collection to final report, into FHIR resources. This will test whether all necessary data elements can be properly

represented. During the transition phase, laboratories are expected to produce both a human-readable PDF report and a structured FHIR-format message. Over time, as the standards mature, the structured data will become the primary format, enabling seamless electronic exchange of genomic results between systems. This gradual and collaborative approach ensures that integration moves forward only once the community agrees on shared, standardized content definitions. This helps to avoid the fragmentation and data silos seen in previous attempts at interoperability.

5 REFERENCES

Bidgood, W. D., Horii, S. C., Prior, F. W., & Van Syckle, D. E. (1997). Understanding and Using DICOM, the Data Interchange Standard for Biomedical Imaging. In Journal of the American Medical Informatics Association (Vol. 4, Number 3). https://doi.org/10.1136/jamia.1997.0040199

Hamosh, A., Scott, A. F., Amberger, J. S., Bocchini, C. A., & McKusick, V. A. (2005). Online Mendelian Inheritance in Man (OMIM), a knowledgebase of human genes and genetic disorders. Nucleic Acids Research, 33(DATABASE ISS.). https://doi.org/10.1093/nar/gki033

Hart, R. K., Fokkema, I. F. A. C., DiStefano, M., Hastings, R., Laros, J. F. J., Taylor, R., Wagner, A. H., & den Dunnen, J. T. (2024). HGVS Nomenclature 2024: improvements to community engagement, usability, and computability. Genome Medicine, 16(1). https://doi.org/10.1186/s13073-024-01421-5

Laboratory Results guidelines, Release 1.1, Jun 2023. (n.d.).

Polak, M., & Cavé, H. (2007). Orphanet Journal of Rare Diseases. Orphanet Journal of Rare Diseases, 2.

Richards, S., Aziz, N., Bale, S., Bick, D., Das, S., Gastier-Foster, J., Grody, W. W., Hegde, M., Lyon, E., Spector, E., Voelkerding, K., & Rehm, H. L. (2015). Standards and guidelines for the interpretation of sequence variants: A joint consensus recommendation of the American College of Medical Genetics and Genomics and the Association for Molecular Pathology. Genetics in Medicine, 17(5). https://doi.org/10.1038/gim.2015.30

Robinson, P. N., Köhler, S., Bauer, S., Seelow, D., Horn, D., & Mundlos, S. (2008). The Human Phenotype Ontology: A Tool for Annotating and Analyzing Human Hereditary Disease. The American Journal of Human Genetics, 83(5). https://doi.org/10.1016/j.ajhg.2008.09.017

Yeh, C. Y., Peng, S. J., Yang, H. C., Islam, M., Poly, T. N., Hsu, C. Y., Huff, S. M., Chen, H. C., & Lin, M. C. (2021). Logical observation identifiers names and codes (Loinc®) applied to microbiology: A national laboratory mapping experience in Taiwan. Diagnostics, 11(9). https://doi.org/10.3390/diagnostics11091564

9.4 Appendix D: Press Release

Cyprus leading in Digital Health with the Workshop

«Integrating Genomics into the EHR – Gen2EHR Implementation Guidelines Workshop»

The National eHealth Authority (NeHA), as the competent authority for eHealth in Cyprus, in collaboration with the Cyprus Institute of Neurology and Genetics and the University of Cyprus, is organizing and funding the workshop **«Integrating Genomics into the EHR – Gen2EHR Implementation Guidelines Workshop»,** which will take place on **29-30 September 2025**, at the Cyprus Institute of Neurology and Genetics in Nicosia.

The workshop is held within the framework of the European Health Data Space Regulation (EHDS), which entered into force in March 2025. Its objective is to present and shape the prototype design of the data flow from the citizen's genome into the Electronic Health Record (EHR), in order to support the secondary use of data for improving healthcare, advancing research, and supporting health policy-making.

The workshop is addressed to healthcare providers, researchers, health policy-makers, physicians, and health professionals. The program includes presentations, discussions, and working sessions with the participation of leading scientists from Cyprus and abroad. The first day (29/09/2025) will feature open sessions, while the second day (30/09/2025) will consist of closed working sessions for invited participants and will conclude with an open session presenting the workshop's outcomes.

At the technical level, the workshop is co-organized by the following projects, which are funded by the European Union:

- Extended EHR@EU Data Space for Primary Use (Xt-EHR) Joint Action, coordinated at the European level by the National eHealth Authority, and
- European Health Data Space for Secondary Use @CY (CY-EHDS-2ND), also coordinated by the National eHealth Authority.

Cyprus at the forefront of Europe in Digital Health and Genomic Data Integration.

Dates: 29-30 September 2025

Location: Amphitheatre, Cyprus Institute of Neurology and Genetics, Nicosia

Participation: Available both on-site and online

Registration: required for on-site participation

Streaming links: 29/09/2025 and 30/09/2025
