# Supplementary material for: Making Genomic Data Actionable in Electronic Health Records: Practical Viewpoint on Standards, Governance, and Implementation
Source: JMIR Med Inform. 2026 Oct 21;14:e99130. doi: 10.2196/99130 (PMC13686338; doi:10.2196/99130)
Supplement: Supplementary file 2 [file medinform-v14-e99130-s001.docx]

| **Step** | **Tool** | **Description** | **Application** | **URL** | **Reference** |
| --- | --- | --- | --- | --- | --- |
| Alignment | BWA-MEM | Burrows-Wheeler Aligner for short-read mapping to reference genome | Both | <https://github.com/lh3/bwa> | ^90^ |
| Alignment | BWA-MEM2 | Optimized version of BWA-MEM for high-performance computing | Both | <https://github.com/bwa-mem2/bwa-mem2> | ^90^ |
| Alignment | DRAGMAP | Illumina’s hardware-accelerated read mapper | Both | <https://github.com/Illumina/DRAGMAP> | ^91–93^ |
| SNV Calling | HaplotypeCaller | GATK’s variant caller for SNPs (single nucleotide polymorphism) and indels (insertion-deletion) | Germline | <https://gatk.broadinstitute.org/hc/en-us> | ^94,95^ |
| SNV Calling | DeepVariant | Deep learning-based variant caller by Google | (DeepVariant: Germline; DeepSomatic: Somatic) | <https://github.com/google/deepvariant>  <https://github.com/google/deepsomatic> | ^91,94,96^ |
| SNV Calling | Strelka | Illumina’s Germline and somatic small variant caller | Both | <https://github.com/Illumina/strelka> | ^97–101^ |
| SNV Calling | mpileup | SAMtools/BCFtools core utility for calculating genotype likelihoods | Both | <https://github.com/samtools/samtools> | ^95,100^ |
| SNV Calling | FreeBayes | Haplotype-based, Bayesian genetic variant detector | Both | <https://github.com/freebayes/freebayes> | ^99,100,102^ |
| SNV Calling | Mutect2 | GATK tool for somatic short variant discovery (SNV and Indels) | Somatic | <https://gatk.broadinstitute.org/hc/en-us> | ^99,101^ |
| SV/CNV Calling | Manta | Structural variant caller for indels, deletions, and translocations | Both | <https://github.com/Illumina/manta> | ^103^ |
| SV/CNV Calling | TIDDIT | Tool for detecting structural variants and large-scale CNVs (Copy Number Variants) | Both | <https://github.com/SciLifeLab/TIDDIT> | ^104^ |
| SV/CNV Calling | CNVkit | Toolkit for copy number variation detection from DNA sequencing | Somatic (primarily) | <https://github.com/etal/cnvkit> | ^105^ |
| SV/CNV Calling | ASCAT | Allele-specific copy number analysis, optimized for tumor purity | Somatic | <https://github.com/VanLoo-lab/ascat> | ^106^ |
| SV/CNV Calling | Control-FREEC | Tool for detection of copy number alterations and allelic imbalances | Both | <https://github.com/BoevaLab/FREEC> | ^107^ |
| Annotation | VEP | Variant Effect Predictor (Ensembl). Determines functional impact of variants on genes and proteins | Both | <https://github.com/Ensembl/ensembl-vep> | ^108,109^ |
| Annotation | SnpEff | Fast annotation and effect prediction for genomic variants | Both | <https://github.com/pcingola/SnpEff> | ^110,111^ |
| Reporting | MultiQC | Aggregates QC reports from multiple tools into a single HTML summary | Both | <https://seqera.io/multiqc/> | ^112^ |
